# Significance of bulky mass and residual tumor—Treated with or without consolidative radiotherapy—To the risk of relapse in DLBCL patients

**DOI:** 10.1002/cam4.2798

**Published:** 2020-01-22

**Authors:** Susanna Tokola, Hanne Kuitunen, Taina Turpeenniemi‐Hujanen, Outi Kuittinen

**Affiliations:** ^1^ Department of Oncology and Radiotherapy Oulu University Hospital Oulu University Oulu Finland; ^2^ Faculty of Health Medicine Department of Oncology Institute of Clinical Medicine Kuopio University Hospital University of Eastern Finland Oulu Finland

**Keywords:** bulky, DLBCL, radiotherapy, residual

## Abstract

Bulky and residual tumor are considered to increase the risk of relapse in diffuse large B‐cell lymphoma (DLBCL) patients. Radiotherapy is conventionally used to reduce the risk, but the evidence is controversial. We performed a retrospective analysis to evaluate the significance of bulky and residual tumor treated with or without radiotherapy in DLBCL patients. We analyzed 312 DLBCL patients treated from 2010‐2017 in Oulu University Hospital. A bulky tumor was detected in 123 patients and 55 of these patients (44.3%) received consolidative radiation therapy (RT) to the bulky tumor. Residual tumor meeting the required criteria was found in 138 (39.3%) patients, and 65 (45.5%) of these patients received consolidative RT to the site of residual tumor. iPET‐CT scans were performed in 102 patients. In multivariate analyses, bulky was an independent risk factor in limited stage patients in progression free survival (HR 6.43 [95%CI 1.609‐25.710]; *P* = .008) not related to International prognostic index (HR 1.35 [95% CI 0.256‐7.124]; *P* = .724) or age (HR 1.62 [95% CI 0.468‐5.638]; *P* = .445). This was not seen in advanced stage patients or in patients with residual tumor. Radiotherapy to the bulky or residual tumor was not able to improve the long‐term PFS of patients. In this study, it appears that performing iPET is the most convincing method in improving evaluation and in finding patients with increased risk of relapse. Evidently, patients with negative iPET will not benefit from including RT in the treatment after metabolic complete response (CR), and patients with primary refractory disease are most likely in the group of positive iPET.

## Graphical abstract

Among patients with limited stage DLBCL, we found a statistically significant effect of bulky tumor to the outcome, but this was not seen in advanced stage disease or in patients with residual tumor. Radiotherapy to the bulky or residual tumor was not able to improve the long‐term PFS and evidently patients with negative iPET won't benefit from including RT in treatment after metabolic complete response. Patients with primary refractory disease are most likely in the group of positive iPET.

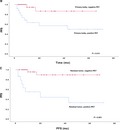

## 1 INTRODUCTION

Diffuse large B‐cell lymphoma (DLBCL) is the most common non‐Hodgkin lymphoma (NHL) in western countries, with an incidence rate of 6.9/100 000 (2011‐2012 in US).^1^, ^2^ The incidence has been increasing in developed regions, but fortunately, cure rates have also improved^3^ from 30%‐40% in the pre‐rituximab era up to 60%‐70% in the rituximab era.^4^, ^5^ Despite developed treatments, there is still a portion of patients whose disease responds to treatments insufficiently or relapses after successful therapy. These patients have poor outcomes, especially when relapse occurs within 12 months after the first‐line treatment.^6^ To avoid excess inefficient therapies while optimizing the outcome of treatment, it would be important to recognize patients with diseases resistant to immunochemotherapy in the early phases of treatment. This provides the ability to change the treatment protocol to salvage therapy as soon as possible when needed and spare patients from unnecessary treatment.

High International Prognostic Index (IPI) scores, the presence of bulky tumor and extranodal diseases are considered as markers of poor prognosis.^8^, ^9^ Tumors with a size of 5‐10 cm have been considered as a cut‐off diameter of bulky mass, and, according to the MInT study, it is most likely closer to 10 cm in the rituximab era. However, the full impact of bulky tumor to prognosis during the rituximab era is not established.^9^

The role of consolidation radiotherapy (RT) in the treatment of DLBCL needs further clarification. Considering the serious long‐term adverse effects of radiotherapy, this is an important issue to resolve in order to avoid unnecessary toxicity. Factors that have been suggested as indications to radiotherapy are primary bulky disease, residual tumor mass after immunochemotherapy and extranodal tumor locality.^9^, ^10^, ^11^, ^12^

This is a retrospective study using the data collected from patient records of Oulu University Hospital including 351 DLBCL patients treated with curative intent from 2010 to 2017. We evaluated the significance of primary bulky tumor and/or residual tumor mass, as well as the impact of radiotherapy to the risk of relapse. Our aim was to find tools to identify early on patients with insufficient response and in need of intensification of treatment.

## 2 METHODS

### 2.1 Patients, staging and treatment

Data were collected from patient records of Oulu University Hospital including 351 DLBCL patients treated between 1/2010 and 11/2017. Seventeen patients with solitary CNS relapses and 22 patients with excessively incomplete data were excluded and after these exclusions 312 subjects were included. An appeal was made to the statement of the Oulu University Hospital ethics committee in this study. A whole‐body computed tomography (CT) was performed in all patients, and bone marrow biopsy and aspiration were performed for 267 patients at the staging. Staging was evaluated according to the Ann Arbor classification. PET‐CT (positron emission tomography‐CT) was performed in 102 patients at some stage of the treatment, and it was interpreted according to the Deauville/Lugano criteria; a Deauville score of ≥4 was regarded as positive.^13^ WHO performance scores were defined between grades 0 and 4, and the IPI scale ranging from 1 to 5 was referenced. Patient characteristics are presented in Table 1.

**Table 1:** Patient characteristics

Variable	Mean or No. of patients (312pt.)	SD or %	Patients treated		Patientsunderwent iPET (102pt.)
			With RT (114pt.)	Without RT (198pt.)	
Gender					
Female	139	45%	48	91	45
Male	173	55%	66	107	57
Age (y)					
<70	175	56%	64	111	76
≥70	135	43%	50	87	26
No data	2	0.6%			
WHO classification					
0	49	16%	19	30	22
1	109	35%	41	68	32
2	60	19%	22	38	28
3	26	8%	10	16	7
4	17	5%	1	16	2
No data	51	16%	21	30	11
IPI					
1	41	13%	16	25	7
2	68	22%	28	40	23
3	65	21%	24	41	33
4	63	20%	11	52	20
5	22	7%	7	15	7
No data	53	17%	3	25	12
Stage					
1	46	15%	18	28	5
2	41	13%	19	22	13
3	57	18%	15	42	25
4	133	43%	42	91	51
No data	35	11%	20	15	8
B‐symptoms					
Yes	162	52%	48	114	65
No	112	36%	45	67	29
No data	38	12%	21	17	8
Treatment protocols					
R‐CHOPx4‐8	122	39%	43	78	50
R‐CHOEPx6‐8	38	12%	12	25	20
R‐CEOPx4‐8	76	24%	39	37	14
Other or no chemotherapy	76	25%	20	58	18

Patients were treated mainly with R‐CHOP regimen (rituximab, doxorubicin, cyclophosphamide, vincristine and prednisolone) ×6‐8 (n = 113) or R‐CEOP (rituximab, epirubicin, cyclophosphamide, vincristine, prednisolone) ×6‐8 (n = 76) with or without the preface therapy. Other treatment protocols included R‐CHOEP (rituximab, doxorubicin, cyclophosphamide, etoposide, vincristine, prednisolone) and R‐CVOP (rituximab, cyclophosphamide, etoposide, vincristine, prednisolone). Intensive treatment eligible patients with relapsed disease were treated by a salvage induction therapy and after sufficient response were consolidated with high‐dose therapy followed by autologous stem cell transplantation (ASCT) (n = 18). Consolidative RT was given to 114 patients (36.5%). The dose of RT was on the average 36 Gy (42.6%) or 40 Gy (42.6%). Three patients did not complete RT.

The response was evaluated through CT scan and bone marrow biopsy in those with initial bone marrow involvement. CT was performed after the fourth, fifth, sixth, or eighth cycle of the treatment, and bone marrow biopsy was performed after the second or fourth cycle. The patient's disease was defined as a primary refractor if there was progression during the treatment, viable lymphoma tissue in biopsy after positive PET‐CT, or relapse within six months after treatment. Follow‐up evaluation was performed at the end of treatment and every three months during the follow‐up for two years and then every six months for five years. Response evaluation was executed by CT/PET‐CT in accordance to the revised International Working Group response criteria^14^ and PET‐CT in accordance with the Deauville/Lugano criteria.^15^ PET‐CT was generally performed after the fourth regimen (59%), fifth regimen (14%) or sixth (15%) regimen of treatment, and seven patients also underwent control PET‐CT after first positive interim‐PET (iPET).

Primary tumor size was measured from the maximal tumor diameter of the largest tumor site with the bulky cut‐off limit considered to be ≥7.5 cm. Cut‐off limit was assessed according to the ROC‐curve (receiver operating characteristic curve). Residual tumor size was measured from the maximal diameter of the widest residual tumor. The cut‐off limit for the residual tumor was evaluated to be ≥1.5 cm after assessing it according to the ROC curve.

Progression‐free survival (PFS) was calculated from the date of the diagnosis to the date of disease progression or disease‐related death or the last follow‐up date. Overall survival (OS) was calculated from the date of diagnosis to the date of death for any reason.

### 2.2 Statistics

All analyses were performed by using IBM SPSS Statistics for Windows & Mac OSX. Survival analyses with corresponding *P*‐values were calculated using the Kaplan‐Meier method with the log‐rank test. Multivariate analyses were calculated using the Cox regression model.

## 3 RESULTS

### 3.1 Prognostic significance of primary bulky disease and consolidative RT

**Figure 1:**
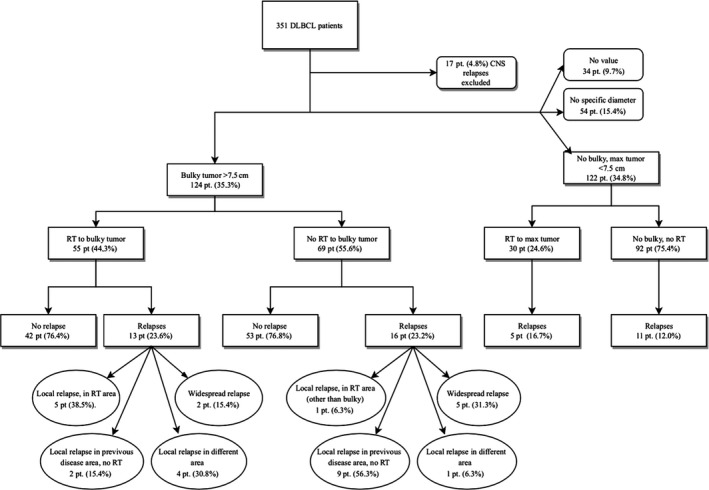
Flowchart, bulky

In the study, 312 patients were analyzed, including 124 patients with primary bulky defined by a cut‐off limit of ≥7.5 cm (Figure 1). Median age was the same when comparing patients according bulky or not; 68 years in patients with vs 67 years without bulky. WHO performance status was worse in the group with bulky tumor, which was detected to be 0‐2 in 70% of patients, compared to 82% without bulky (*P* < .001). IPI scores were higher in patients with bulky, being over 2 in 68% vs 46% without bulky (*P* = .011), and the stage of the disease was over 2 with 73% of patients with and 53% without bulky (*P* = .001).

**Figure 2:**
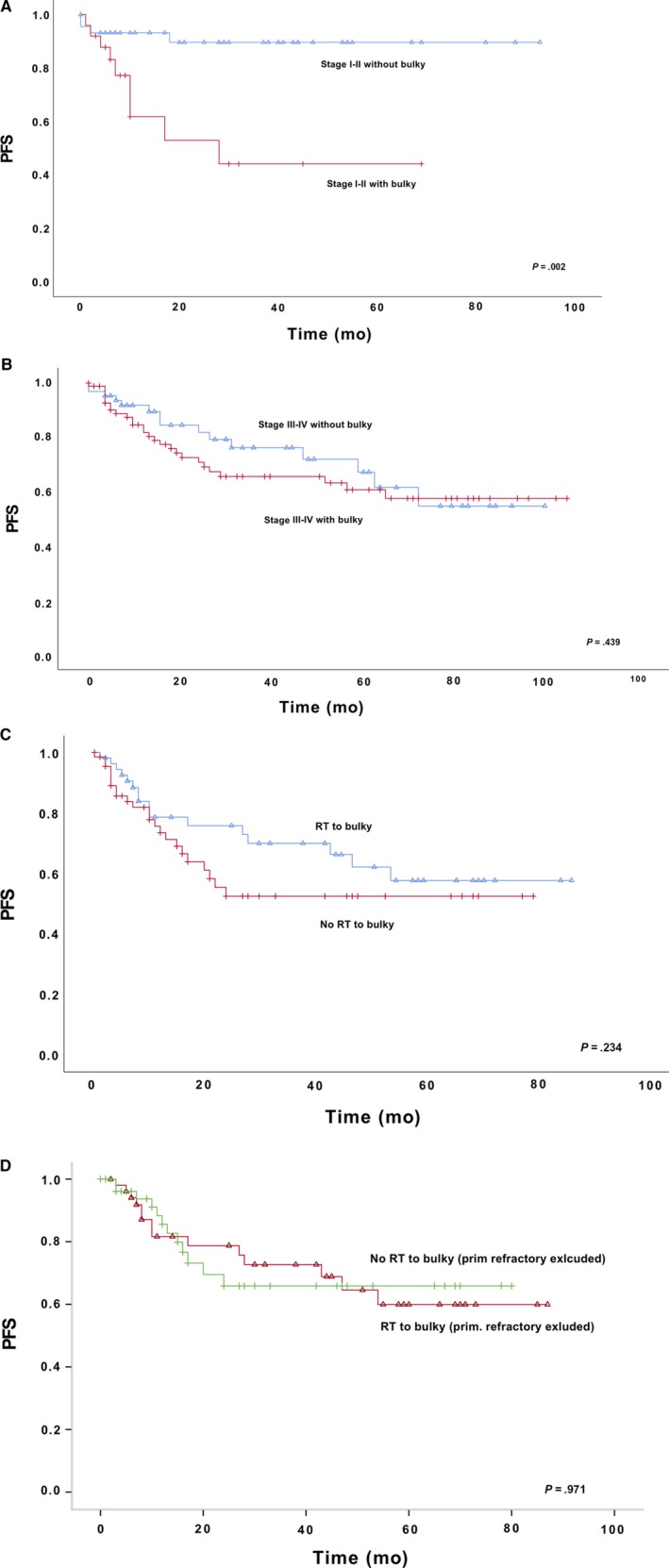
A, bulky, limited stage B, bulky, advanced stage C, bulky, RT or not to bulky D, RT, primary refractory excluded

In the entire study population, bulky tumor had no prognostic impact and in multivariate analysis it was not statistical significant (HR 1.70 [95% CI 0.93‐3.12]; *P* = .085), IPI (HR1.5 [95% CI 0.63‐3.55]; *P* = .32), stage (HR 0.96 [95% CI 0.38‐2.43]; *P* = .933) and age (HR 1.74 [95%CI 0.99‐3.06]; *P* = .055). However, after splitting the data according to the stage into limited (I‐II) or advanced stage (III‐IV), the existence of bulky tumor was associated with inferior prognosis in limited stage disease. For patients with limited stage disease, the 2‐ and 5‐year PFS rates were both 90% without bulky, and the corresponding numbers for those with bulky tumor were respectively 53% and 44% (*P* = .002). For patients with advanced stage the 2‐year PFS was 79% and 5‐year 55% without bulky, and with bulky tumor they were respectively 66% and 58% (*P* = .439). (Figure 2A) In multivariate analyses in limited stage patients, the bulky was an independent risk factor in progression‐free survival (HR 6.43 [95%CI 1.6‐25.7]; *P* = .008) not related to International prognostic index (HR 1.35 [95% CI 0.26‐7.12]; *P* = .724) or age (HR 1.62 [95% CI 0.46‐5.63]; *P* = .445). This was not seen in advanced stage patients.

Consolidative RT was administered to the bulky tumor site in 55 (44.3%) patients with bulky tumor. A trend was seen in which RT was able to delay relapse in the whole population; but after excluding primary refractory patients, this difference vanished. When consolidative RT was targeted to the bulky tumor, the 2‐year PFS was 76% and 5‐year 58%; without consolidative RT, the 2‐year and 5‐year PFS was 53% among patients with bulky tumor (*P* = .234) (Figure 2C). After excluding patients with primary refractory disease, the 2‐year PFS was 78% with RT to bulky and 66% without RT, and the 5‐year PFS was 60% when treated with RT and 66% without RT (*P* = .971) (Figure 2D).

### 3.2 Prognostic significance of residual tumor mass and consolidative RT

**Figure 3:**
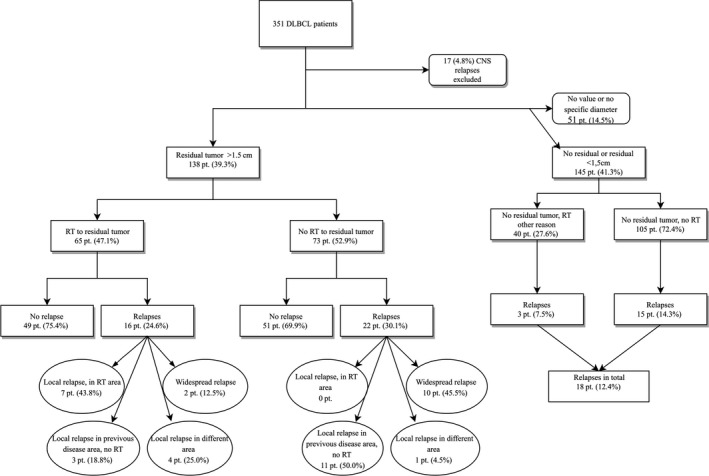
Flowchart, residual tumor

The residual tumor was assessed to meet the criteria in 138 (39.3%) of the patients. Median age was 65 years among patients with a residual vs 69 years without the residual tumor (Figure 3). WHO status was lower in the residual tumor group, being 0‐2 in 85% of patients compared to 66% in those without (*P* = .004). IPI scores over 2 were detected in 66% of the patients with and in 50% of patients without a residual (*P* = .240), and the stage was over 2 in 68% of the patients with and 54% of patients without residual tumor (*P* = .036).

**Figure 4:**
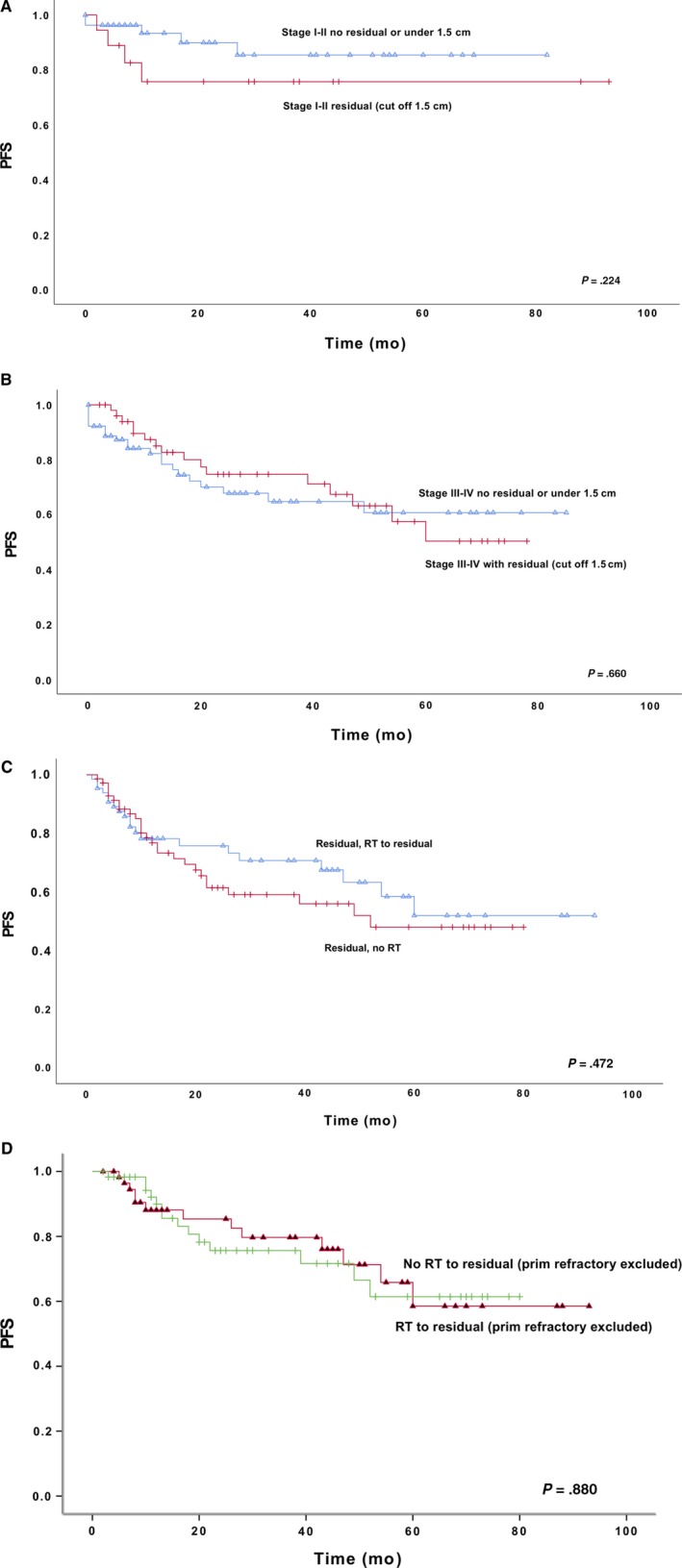
A, Residual tumor limited stage, B, advanced stage, C, residual tumor with or without RT, D, residual tumor ± RT, primary refractory excluded

Residual tumor was not statistically significant in this study. Among patients with limited stage disease and without residual tumor (or a residual tumor under 1.5 cm), the 2‐year PFS was found to be 90% and the 5‐year PFS 85%; with explicit residual tumor, the 2‐year and 5‐year PFS was 76% (*P* = .224) (Figure 4A). In patients with advanced stage disease without residual, the 2‐year PFS was 68% and 5‐year 61%; with residual, the 2‐year was 75% and 5‐year was 50% (*P* = .660) (Figure 4B). In multivariate analyses, residual tumor was either statistical significant (HR 1.03 [95%CI 0.55‐1.92]; *P* = .925) compared to IPI (HR 2.15 [95%CI 0.82‐5.69]; *P* = .122), age (HR 1.48 [95%CI 0.81‐2.71]; *P* = .201), or stage (HR 1.30 [95%CI 0.45‐3.79]; *P* = .631).

Of 138 patients with a residual tumor, 65 (45.5%) received consolidative RT to the site of residual. Also, within patients with residual tumor, we were able to see a trend of a delay of relapses after RT, but this difference vanished after excluding patients with primary refractory disease. The 2‐year PFS was found to be 73% and 5‐year 52% of those treated by RT, while in those without consolidative RT to the site of residual, the 2‐year PFS was 61%, and 5‐year was 48% (*P* = .472) (Figure 4C). After the patients with primary refractory disease were excluded from this data, the 2‐year PFS was 85% when treated with RT to the residual site and 76% if not, and the 5‐year PFS was 59% with RT and 61% without (*P* = .880) (Figure 4D).

In addition, we analyzed percentual decrement of the widest tumor diameter. In PFS statistics, we were not able to see any statistical significance or linear progression between percentual decrement and the risk of relapse.

### 3.3 Prognostic significance of interim PET‐CT

iPET was performed in 102 patients. The median age was 64 years among patients with iPET and 72 years without iPET. WHO performance status was lower within the iPET group, being 0‐2 in 80% of patients compared to 66% in patients without iPET (*P* = .040). IPI scores over 2 were detected in 65% of patients in the group of iPET and 51% in patients without (*P* = .091), but the stage was over 2 with 75% of patients with iPET and 55% without iPET (*P* = .004).

**Figure 5:**
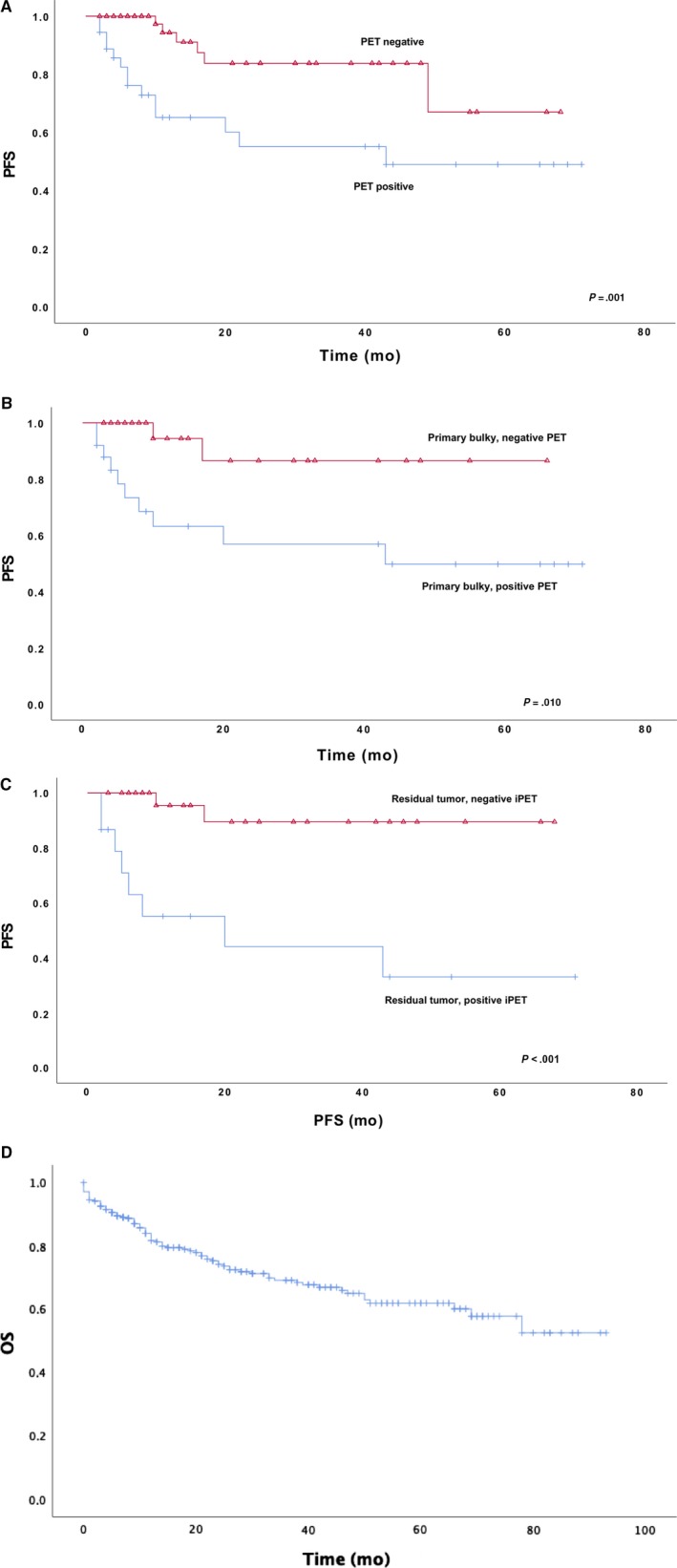
A, PFS iPET positive vs negative B, primary bulky according iPET C, residual tumor according iPET 5 D, OS, all patients (n = 312)

iPET was positive in 36 patients (35%) and negative in 66 patients (65%). IPET was an efficient method in differentiating prognostic groups. The 2‐year PFS in the group of patients with positive iPET was 55%, and the 5‐year was 49%. With negative iPET, corresponding numbers were 84% and 67% (*P* = .001) (Figure 5A). Twenty‐one patients (33%) with negative iPET and 10 patients (29%) with positive iPET were treated with RT after chemotherapy. Prognostic variables of patients did not differ between groups that did or did not receive RT.

Among patients with primary bulky tumor and residual tumor, the PFS was significantly better within the group of negative iPET. The 2‐year PFS was 87% among patients with bulky and negative iPET vs 57% among those with positive iPET, and the 5‐year PFS was 87% among patients with negative iPET vs 50% with positive iPET (*P* = .010). Corresponding PFS with residual tumor was 90% at 2‐year with negative iPET vs 44% at 2‐year with positive iPET, and the 5‐year PFS was 90% with negative iPET vs 33% with positive iPET (*P* < .001). When analyzing the relapses in the negative iPET group, 7 out of 66 patients had disease relapse during the follow‐up, and all of these relapses were either outside of the primary tumor location, in the field of radiotherapy or cases in which the disease was widespread and accordingly not preventable with RT.

## 4 DISCUSSION

In this retrospective analysis, we found that initial bulky tumor was related to adverse prognosis in DLBCL patients with a limited stage disease but not in those with an advanced stage disease. Radiotherapy to the primary bulky or to the residual tumor seemed to delay disease progression in the whole population, but after exclusion of patients with primary refractory diseases, this phenomenon vanished, and it was not associated with improvement of long‐term outcome. We also found no statistically significant association between residual tumor mass and risk of relapse. In contrast, a positive iPET scan was associated with increased risk of relapse.

DLBCL is the most common NHL in western countries, and most patients are cured of their disease.^1^, ^2^ Despite development in treatments, there is still a proportion of DLBCL patients succumbing to their disease, so clearly there is a need to improve the treatment results.^4^ On the other hand, most common consolidation therapies, radiation and autologous stem cell transplantation also increase long‐term toxicities of the therapy and thus should be reserved only for patients with insufficient response to treatment.^7^, ^16^, ^17^, ^18^, ^19^ To optimize DLBCL treatment, we should be able to stratify patients into different prognostic groups already during the time of the primary diagnosis and later tailor the optimal therapy according to the response. In this work, we evaluated the prognostic impact of the primary bulky tumor, residual tumor mass and iPET, as well as the impact of consolidation radiotherapy to the risk of relapse.

The tumor response after immunochemotherapy has traditionally been assessed with a CT scanaccording to the Cheson criteria published in 1999.^20^ In clinical practice, it is known that such criteria do not correlate sufficiently with the risk of relapse. Some patients with radiological partial response (PR) are actually already cured, and some with complete response (CR) will suffer relapse. For these reasons, the revised Cheson criteria was published in 2007 and metabolic tumor response assessed with PET‐imaging was taken into account after the therapy.^14^ Significance of PET‐negative residual masses after chemotherapy is unclear and controversial. Some results suggest improved outcome to patients with radiologic CR, whereas others show no difference between outcome regardless of PET‐negative residual mass.^21^, ^22^, ^23^ The significance of residual tumor was not statistically significant in this study but the 2‐year PFS difference according residual was 90% vs 76% in limited stage patients. The difference diminished after follow‐up, but it still remained after five year (85% vs 76%). However, according to iPET results, our findings imply that most of the residual masses represent nonviable scar tissue and should not be regarded as treatment failure. These results strengthen the importance of PET‐imaging to distinguish factual residual disease from inactive residual masses.

In this study, 102 patients underwent iPET, and in this population it was shown that PET negative residual mass did not predict relapse of disease. Among patients with positive iPET, 28% relapsed within the follow‐up time, while the risk of relapse was 11% in the group of patients with negative iPET (*P* = .029). The prognosis of patients with negative iPET was excellent, which is partially explained by patient characteristics. Before 2015, PET CT was performed in patients who were considered as candidates to ASCT, so patients were younger and had better performance status. However, PFS was favorable in the group of negative iPET (*P* = .001), and this was also seen after splitting data according to limited and advanced stage diseases. Most patients were also cured regardless of positive iPET, and this raises the issue of challenges of the high false positive rate of iPET.

According to the MInT Trial, the existence of bulky tumor is associated with higher relapse rate in limited stage DLBCL.^24^ In this study, we were able to confirm the findings of the MInT study among patients with limited stage disease. However, our results suggest that the prognosis of limited stage disease with a bulky tumor seems to be similar to that of advanced stage disease. These results raise the question of whether we should assess patients with limited stage disease presenting with a bulky tumor equal to patients with an advanced stage disease. In contrast, in advanced stage disease, the bulky tumor had no independent prognostic value. It is conceivable that with heavy tumor load and widespread disease, the significance of single tumor size diminishes and overall disease burden is more meaningful for the prognosis.

This study PFS varies within patients with bulky tumor according to RT to the bulky tumor (2‐year PFS 76% vs 53%), but it diminished during follow‐up (5‐year PFS 58% vs 53%), and this was not seen when and after patients with primary refractory disease were excluded (2‐year PFS 78% vs 66%). This same delay was also seen among patients with residual mass and RT to residual, in which the 2‐year PFS was 76% vs 61%, and the 5‐year PFS was 52% vs 48%, in which case it also vanished after exclusion of primary refractory diseases. All these differences also diminished and lost their value if only patients who underwent iPET were included. Our results imply that it is conceivable that RT delays the relapse of disease but has only a minor impact on the cure rates. This same phenomenon has been detected in the long‐term analysis of the Southwest Oncology Group Study S8736 with limited‐stage DLBCL in the pre‐rituximab era.^22^ In addition, we were not able to see any differences according to RT after exclusion of patients with primary refractory diseases.

The role of radiotherapy is ambiguous, and it is difficult to make explicit conclusions from literature. According to the MInT study, RT was shown to be beneficial in young patients with a bulky tumor and IPI < 2, but with IPI ≥ 2, it is unclear and in need of further study.^9^ Results of patients over 60 years seem to be in line with results of younger patients. RT has no benefit for patients with local disease,^11^ but according to a RICOVER‐60 trial with bulky tumor, it may be able to improve the outcome.^12^ According to Pfreundschuh et al, results from OPTIMAL > 60 study, it seems that RT can be spared from patients with bulky but negative iPET after chemotherapy without compromising the results.^12^ In Norway, researchers reported improved outcome among patients with any residual mass and RT vs no RT, but it was not evident in patients who were considered high‐risk (IPI 4‐5).^25^ Recently published results of randomized study found that R‐CHOP alone is not inferior compared to R‐CHOP + RT among patients with limited‐stage disease and without bulky tumor. This study included iPET as an evaluation method for all patients.^10^

In this study, based on locations of relapses, relapses among patients with negative iPET were not preventable with RT. On the basis of these findings, patients with negative iPET will not benefit from RT being included in the treatment after metabolic CR response, and patients with primary refractory disease are most likely in the group of positive PET. RT is a toxic treatment, and it has major delayed adverse effects and is probably unnecessary in the group of patients with negative iPET. According to these results and previous literature, it seems that iPET may be able to assist in distinguishing patients who may need intensification of treatment, and for patients who may not tolerate intensive salvage treatments or ASCT, RT is a legitimate treatment option. RT may be able to prolong the length of response, although the impact toward a permanent cure is not convincing. In this study, we found minor effect of RT which was not statistically significant. If there is real benefit from consolidative radiotherapy it would be probably limited to patients with PET positive residual mass. However, the number of cases in this study is limited and no definitive conclusions can be reached regarding the role of radiotherapy further studiesare needed in this field.

Patients with solitary CNS relapses were excluded from this study. Solitary CNS relapses are thought to arise from CNS dissemination of lymphomas before any therapy and it is a situation that could not be prevented by consolidation radiotherapy to the primary tumor site. Patients with risk of CNS relapses should already be identified at the time of diagnosis, and CNS prophylaxis should be combined with the conventional treatment regimen at the early stage.

The data in this study were comprehensive, and patients were treated with comparatively homogenous treatment protocols. Data included detailed clinical information, and iPET coverage was 33%. The strengths of this study were that we also excluded CNS relapses to gain insight into accurate outcomes among patients who are treatable with protocols of systemic disease. The weakness of this study is its retrospective view, and it is desirable that prospective studies are made specifically to find preferable significance of iPET and RT.

In conclusion, this study repeated the results of the MInT study concerning the adverse effects of bulky tumor to the outcome in the limited stage DLBCL. The prognosis of these patients was analogous to advanced stage disease. In contrast, in advanced stage disease, the value of the bulky tumor vanished, and the existence of residual tumor was not able to predict relapse. These results strengthen the importance of PET in the evaluation of response in differentiating active tumors from inactive residual masses, but a clinician should still be aware of the high rate of false positive PET findings. Whether transplant‐eligible patients with iPET positive residual mass should receive consolidation radiotherapy or proceed straight to second line induction therapy and ASCT needs to be addressed in future studies.

## CONFLICTS OF INTEREST

The authors have no conflict of interest to report.

## Data Availability

The data that support the findings of this study are available on request from the corresponding author. The data are not publicly available due to privacy or ethical restrictions.
